# Unraveling the hidden conditions in NiOOH for electrocatalytic oxidation of methanol to formaldehyde with unity Faraday efficiency and selectivity

**DOI:** 10.1039/d6sc00905k

**Published:** 2026-03-23

**Authors:** Zixuan Ma, Yuling Yuan, Ze Lv, Yimeng Ma

**Affiliations:** a College of Chemistry and Chemical Engineering, Donghua University Shanghai 201620 China yimeng.ma@dhu.edu.cn

## Abstract

Nickel oxyhydroxide (NiOOH) has been widely applied in electrocatalytic water splitting. Limited by the sluggish kinetics of the oxygen evolution reaction, the electrocatalytic oxidation of methanol has emerged as an alternative reaction. Methanol oxidation to higher-value-added formaldehyde is of great significance in organic synthesis. However, the selectivity of formaldehyde remains ambiguous, whereas formic acid, as a further oxidised product, has been frequently reported, raising the question of whether NiOOH enables selective formaldehyde formation. This study investigates the reaction pathway for formaldehyde formation during methanol oxidation in the presence of NiOOH electrocatalysts. The ∼100% selectivity and Faraday efficiency for formaldehyde formation require a low applied potential and high concentration of methanol. *Operando* spectroelectrochemical techniques are employed to reveal the mechanism of formaldehyde formation. Low potential facilitates the formation of NiOOH(3+) as the oxidation species; additionally, 90% methanol prohibits the activation of the second C–H bond, avoiding overoxidation to formic acid. H/D isotope exchange measurements indicate that methanol oxidation is sensitive to the C–H bond activation, which is the primary reason for unreported formaldehyde formation and overoxidation to formic acid in the literature. Therefore, this study reveals the previously overlook conditions for NiOOH to oxidise methanol to higher-value formaldehyde, thus enabling further application in formaldehyde production on a practical scale.

## Abstract 2

This study reveals that the electrocatalytic oxidation of methanol to formaldehyde using NiOOH requires a low applied potential and high concentration of methanol, associated with the control of the 2nd C–H bond activation in the methanol molecule.

## Graphical abstract

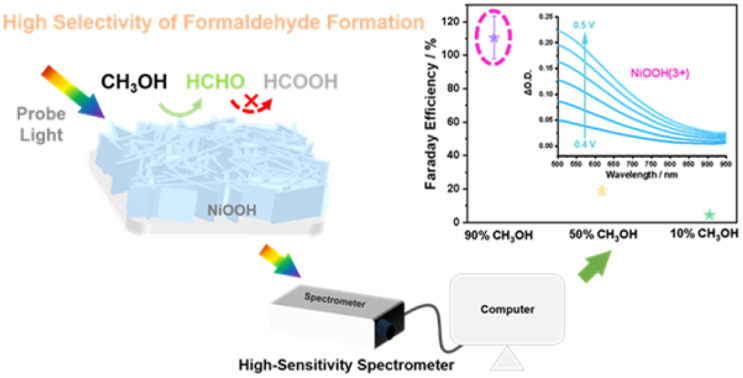

## Introduction

The electrochemical oxidation of methanol is a versatile application in both electrocatalytic synthesis and fuel cell energy conversion.^1–5^ As one of the most important chemical commodities, the greatest challenge in the efficient use of methanol is controlling the oxidation steps towards value-added intermediate products, such as formaldehyde, widely used in resins, plastics, and coatings industries.^6,7^ The market demand for formaldehyde has exceeded 10^7^ tonnes per year,^8^ clearly indicating its importance in the chemical industry. The traditional method of methanol oxidation to formaldehyde is based upon a silver method where high temperature and pressure are required for the production of formaldehyde in ∼90% yield and ∼95% selectivity.^9,10^ The synthetic conditions employed for formaldehyde production also cause health and safety concerns on an industrial scale. In addition, formaldehyde often undergoes overoxidation, producing formic acid as a byproduct, clearly demonstrating the challenge of meeting the green chemistry requirement for synthesising formaldehyde from methanol.

Heterogeneous electrocatalytic oxidation of methanol offers an alternative approach for the synthesis of formaldehyde. The electrocatalytic conditions for the synthesis provide a more facile environment in an aqueous medium, without high temperature or pressure, significantly lowering the risk in industrial chemical production.^11^ In addition, electrocatalysis enables precise control of the reaction conditions to manipulate the reaction pathway, and therefore, the selectivity. This advantage, in principle, enhances the efficiency and green-chemistry efficacy of formaldehyde synthesis, clearly demonstrating the promise of electrocatalysis for the synthesis of important chemical commodities.

To achieve efficient electrocatalysis in organic synthesis, electrocatalysts are pivotal. Precious metals or their oxides are conventionally used as electrocatalysts due to their high efficiency.^12–16^ However, the use of these materials greatly hinders the applicability of electrocatalysis, primarily due to their high cost and limited abundance. In addition, many of the materials suffer from deactivation during organic catalysis due to the generation of carbon monoxide as the byproduct of organic oxidation, which challenges the overall feasibility of using electrocatalysis for value-added and large-scale organic synthesis.^17^

Among the range of electrocatalysts available, earth-abundant metal oxides are particularly promising for active and efficient electrocatalytic oxidations.^18^ Nickel oxyhydroxide (NiOOH) has been extensively investigated in electrocatalysis of water oxidation,^19–21^ organic oxidation,^7,22,23^ biomass valorization^24,25^ and plastic waste reforming.^26,27^ These oxidations are the perfect demonstration of NiOOH as a versatile electrocatalyst with multiple applications, promising its potential for large, industrial-scale use. Despite the promising features of NiOOH, unresolved issues remain in organic oxidation, especially in alcohol oxidation. The most important issue is controlling selectivity in alcohol oxidation to the corresponding aldehyde or carboxylic acid. In methanol oxidation, the oxidation product has been reported primarily as formic acid, with high selectivity and Faraday efficiency.^28–34^ However, formaldehyde as the intermediate molecule has not been a significant focus. One possible reason is that the electrocatalytic conditions are too strong, leading to overoxidation of formaldehyde. For example, an applied electrochemical potential of 1.56–1.71 V_RHE_ was used for methanol oxidation to formaldehyde. Such strong potential was able to drive water oxidation to generate molecular oxygen, making it difficult for formaldehyde to remain unoxidised.^35^ Besides the applied potential range, the concentration of methanol for electrocatalysis has also been reported over a range from 0.4% to 6% (methanol/water, v/v%).^29,36,37^ The different concentrations directly result in variations in the Faraday efficiency and selectivity for formaldehyde, possibly due to concentration-dependent kinetic control. However, these variations in electrocatalytic conditions and the mechanisms underlying the selectivity remain poorly understood. In addition, the conditions that enable the highly selective and efficient synthesis of formaldehyde have not been reported to date. Therefore, it is crucial to ensure the electrocatalytic conditions for formaldehyde synthesis and to understand the reactions that control the selectivity and overoxidation of formaldehyde, which is the key focus of this study.

In this study, the electrocatalytic oxidation of methanol using NiOOH was investigated to reveal the conditions that drive ∼100% selectivity for formaldehyde formation and the reaction mechanism. The selectivity has been found to depend on methanol concentration and applied potential. A methanol concentration above 90% and a rather narrow potential range around the onset were key to ∼100% selectivity for formaldehyde formation, which is reported here for the first time and has been overlooked in the literature. Outside the range of these conditions, formic acid was observed as the product of the overoxidation of formaldehyde. Spectroelectrochemical (SEC) techniques were employed to investigate the kinetics of charge carriers in NiOOH, responsible for ∼100% formaldehyde formation and overoxidation, respectively. Such a technique has been proven to be a powerful tool for investigating charge-carrier dynamics and reaction mechanisms in photo(electro)chemical and electrochemical water splitting,^21,38–42^ organic oxidation,^43,44^ and environmental catalysis.^45,46^ The reaction pathways for formaldehyde formation and overoxidation were also determined using H/D isotope exchange, as assessed by spectroscopic assays. These results provide a detailed description of the conditions required for ∼100% formaldehyde formation *via* methanol oxidation by NiOOH, which have been greatly overlooked, and elucidate the transformation of methanol to formaldehyde under these conditions, as well as formic acid under overoxidation conditions.

## Results and discussion

The NiOOH electrode was electrochemically deposited on FTO substrates using a previously reported method.^21^ The surface morphology of NiOOH, as observed by field emission scanning electron microscopy (FE-SEM), consisted of stacked nanosheets (Fig. S1). No diffraction peaks corresponding to NiOOH were observed in the X-ray diffraction (XRD) data (Fig. S2), attributed to the amorphous structure of NiOOH, also consistent with our previous report and literature.^21,47^ Additionally, we observed that the structure of the prepared NiOOH is independent of the deposition substrate, exhibiting an amorphous structure regardless of whether it is deposited on FTO or carbon paper surfaces. The X-ray photoelectron spectroscopy (XPS) shows that the oxidation state of as-prepared NiOOH was Ni^2+^ with mixed NiO and Ni(OH)_2_ phases (Fig. S3).^21,48,49^ Raman spectroscopy of the as-prepared NiOOH electrode shows characteristic peaks for γ-NiOOH phase (473 cm^−1^) and β-NiOOH phase (554 cm^−1^) (Fig. S4).^21,50^ We are aware that the bulk of the NiOOH has been determined to be amorphous. However, the surface of NiOOH has shown a clear γ -NiOOH and β-NiOOH phases. As Raman spectroscopy is a surface characterization technique, the surface structure is highly likely to be regulated at the electrode–electrolyte interface, in agreement with the literature on similar electrochemically deposited NiOOH electrodes.^50–52^

### Identifying the selectivity and Faraday efficiency of formaldehyde formation

**Fig. 1:**
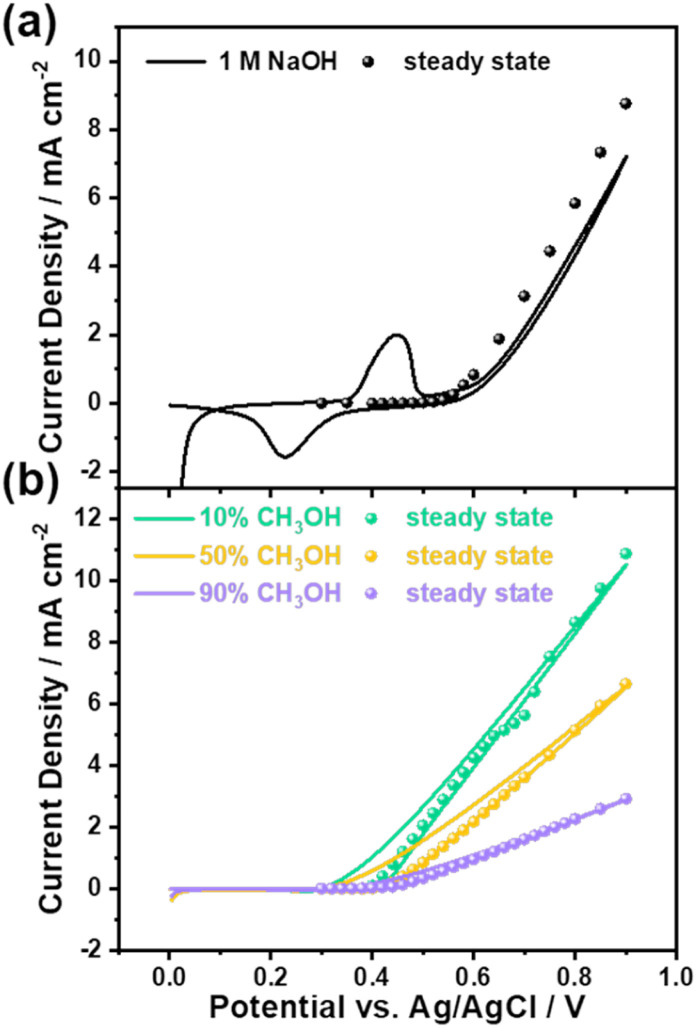
(a) Cyclic voltammetry of electrochemical water oxidation (1 M NaOH, black line) on the NiOOH electrode and the corresponding steady-state current density (black sphere). (b) Cyclic voltammetry of electrochemical methanol oxidation (10% CH_3_OH + 90% 1 M NaOH, green line; 50% CH_3_OH + 50% 1 M NaOH, orange line; 90% CH_3_OH + 10% 1 M NaOH, purple line) on the NiOOH electrode and the corresponding steady-state current density (sphere). More CV data as a function of methanol concentration are given in the SI Fig. S5. Scan rate: 10 mV s^−1^.

Fig. 1 shows the cyclic voltammetry (CV) of the NiOOH electrode oxidising methanol from 0% to 90% in 1 M NaOH (methanol: water, v/v%). Applied potentials are reported against the Ag/AgCl reference electrode without the conversion to the reversible hydrogen electrode (RHE), as the conversion to RHE in highly concentrated organic aqueous solutions might not be accurate.^53^ No *iR* correction was carried out in order to align the optical amplitude in the SEC measurements to the absolute electrochemical current, making the results more meaningful for real-world industrial applications.^54^ In NaOH electrolyte without methanol, only water oxidation occurred on NiOOH with a pair of reversible peaks at ∼ 0.45/0.23 V_Ag/AgCl_ observed in the cyclic voltammetry (Fig. 1a). The redox peak decreased to zero under steady-state conditions, suggesting that these oxidation and reduction processes involved a non-faradaic surface charging process of NiOOH(2+) to NiOOH(3+) because there was no charge transfer across the NiOOH-electrolyte interface. Consequently, the net current was zero at the steady-state condition. This phenomenon is consistent with our previous study and literature.^21,55^ In contrast to water oxidation, methanol oxidation by NiOOH did not exhibit the redox process of NiOOH(3+)/NiOOH(2+) when the onset potential cathodically shifted from 0.5 V_Ag/AgCl_ (NaOH) to 0.4 V_Ag/AgCl_ (methanol/NaOH), as shown in Fig. 1b and S5, suggesting that the electrochemical current observed in the presence of methanol was associated with the oxidation of methanol before water oxidation. This is in agreement with a recent study of methanol oxidation using NiOOH electrocatalysts.^56^ We and others have previously reported that NiOOH(4+), formed after the onset potential of water oxidation (0.5 V_Ag/AgCl_), was the active species responsible for water oxidation after the redox process of NiOOH(3+/2+) at 0.45 V_Ag/AgCl_.^20,57^ Compared to water oxidation, methanol oxidation occurred before the onset potential of water oxidation, suggesting that NiOOH(3+) is an important active species in methanol oxidation, warranting the identification of oxidation products and kinetic analyses as discussed below.

**Fig. 2:**
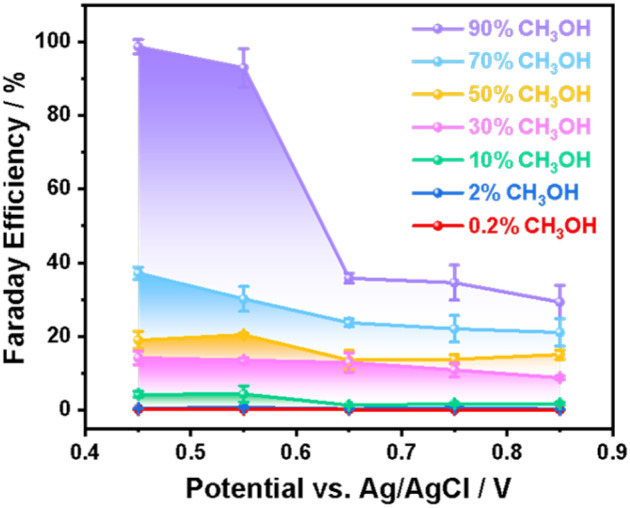
Faraday efficiency of formaldehyde formation on the NiOOH electrode *via* electrocatalytic oxidation of methanol at different concentrations as a function of applied potentials.

Following the electrochemical results of methanol oxidation, we turn to identify the oxidation products and to determine the selectivity. Fig. 2 shows the Faraday efficiency of methanol oxidation to formaldehyde by NiOOH as a function of methanol concentration and applied potential. Spectrophotometric method was employed using 4-amino-3-hydrazino-5-mercapto-1,2,4-triazole (AHMT) to identify the characteristic absorption only with formaldehyde rather than formic acid (Fig. S6), which has been reported previously by our group and others (details given in the SI and Fig. S7).^43,53,58,59^ It is striking that ∼92–98% Faraday efficiency of methanol oxidation to formaldehyde on the NiOOH electrode requires high concentration of methanol (*i.e.* ≥ 90%) and a rather narrow potential range between 0.45 V_Ag/AgCl_ and 0.55 V_Ag/AgCl_. Outside the range of these conditions, the Faraday efficiency decreased sharply with increasing applied potential or H_2_O concentration (*i.e.*, decreasing methanol concentration). We are aware that the nearly unit Faraday efficiency corresponds to almost ∼100% selectivity for formaldehyde formation, as all charges participated in the oxidation of methanol. However, the lower Faraday efficiency by either higher applied potential or lower methanol concentration suggests that oxidation charges are partly involved in the oxidation of methanol to form formaldehyde. The rest of the charges either continued to oxidise formaldehyde or instead oxidised other substrates, such as water molecules or hydroxide ions. The former is considered an overoxidation pathway that decreases both the Faraday efficiency and the selectivity for formaldehyde formation. The latter only decreased the Faraday efficiency; however, the selectivity of formaldehyde generation would remain high. Therefore, it is pivotal to distinguish the primary cause of the lower Faraday efficiency and to identify the selectivity under these non-unity faradaic conditions.

**Fig. 3:**
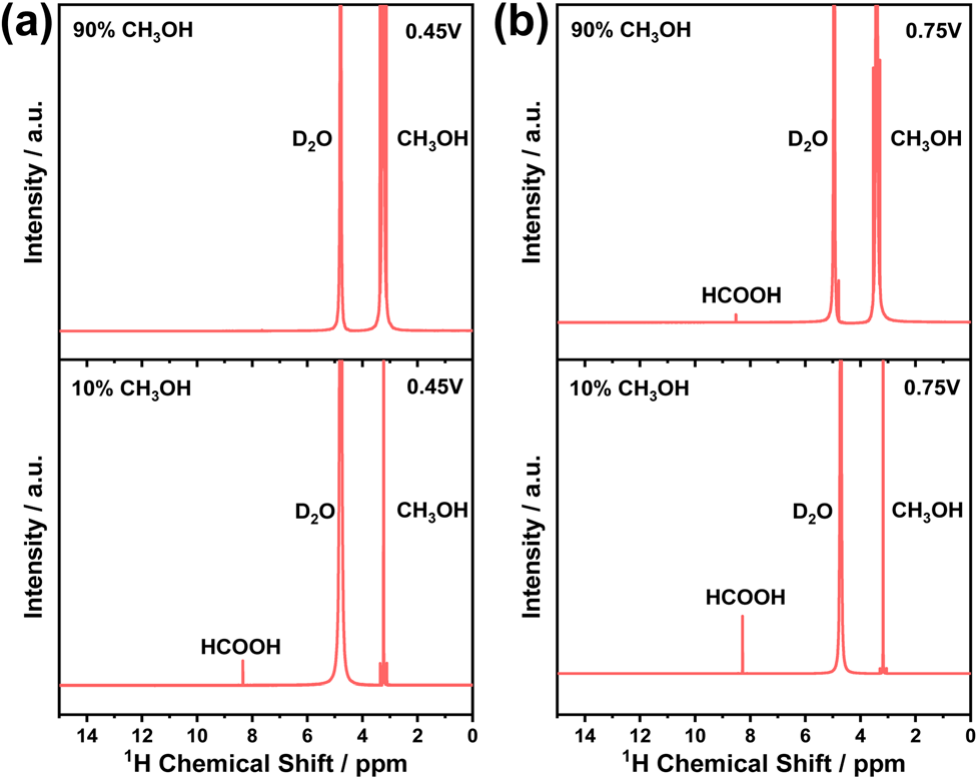
(a) The ^1^H nuclear magnetic resonance spectra of oxidation products recorded at 0.45 V_Ag/AgCl_ in 90% (upper) and 10% (lower) methanol. (b) The ^1^H nuclear magnetic resonance spectra of oxidation products recorded at 0.75 V_Ag/AgCl_ in 90% (upper) and 10% (lower) methanol. All electrocatalytic reactions were carried out over 54 hours to accumulate the oxidation products for the NMR characterizations.

We now turn to the non-faradaic process of electrocatalytic methanol oxidation by NiOOH as a function of applied potential and methanol concentration. Fig. 3 shows the identification of formic acid using ^1^H nuclear magnetic resonance (NMR) spectroscopy. The assay for product identification was first carried out at a low applied potential (0.45 V_Ag/AgCl_) in 10% and 90% methanol electrolytes. Fig. 3a (upper) shows that no formic acid was formed in the 90% methanol electrolyte at 0.45 V_Ag/AgCl_, according to the ^1^H NMR result. Together with the ∼100% Faraday efficiency for formaldehyde formation under the identical conditions in Fig. 2, this NMR result further consolidates the high selectivity of formaldehyde formation, as all oxidation charges in NiOOH participated in the methanol oxidation, and the formaldehyde was the only product from methanol. The OER, on the other hand, is clearly ruled out simply because the applied potential was insufficient to drive the OER, as seen in Fig. 1a.

In contrast to the formaldehyde formation in high methanol concentration and low potential, formic acid was characterised in low methanol concentration (10%) under the same low potential (0.45 V_Ag/AgCl_) shown in Fig. 3a (lower). This observation of formic acid under such conditions is broadly consistent with literature reports.^29,30,36^ In 10% methanol concentration, the characteristic peaks at 8.3 ppm in ^1^H NMR were clearly observed,^29^ suggesting that the process of methanol oxidation was unable to terminate at the formaldehyde step. The low Faraday efficiency (∼4%) measured under these conditions (10% methanol/0.45 V_Ag/AgCl_, shown in Fig. 2) is attributed to the further oxidation of formaldehyde by NiOOH. Further oxidation proceeded in the electrolyte, where water molecules were the predominant species, suggesting that water molecules participated in the oxidation of formaldehyde, although the applied potential was unable to oxidise water molecules, as no OER current was observed in 1 M NaOH (Fig. 1a).

The oxidation products measured under high potential (0.75 V_Ag/AgCl_) as a function of methanol concentration are now considered. Unlike in low-potential conditions, applying 0.75 V_Ag/AgCl_ to NiOOH results in a significant OER current in 1 M NaOH electrolyte (Fig. 1a). In the presence of methanol, not only was formaldehyde the oxidation product (Fig. 2), but formic acid was also detected, regardless of methanol concentration, as shown in Fig. 3b. Although OER was observed without methanol, oxygen was only detected during oxidation at concentrations below 2% methanol in Fig. S8, suggesting that in the 10% and 90% methanol electrolytes, all oxidation charges in NiOOH oxidised methanol molecules rather than water molecules, consistent with the literature on the formic acid synthesis when high anodic potential was applied to the NiOOH electrode.^35^ Apparently, the applied potential significantly increased the energetics of the oxidation charges, thereby inducing the sequential oxidation of methanol to formaldehyde, and subsequently to formic acid.

We turn to discuss the selectivity of formaldehyde electrocatalytically oxidised from methanol by NiOOH. Electrocatalytic methanol oxidation has often been employed to synthesise formic acid with high yield and selectivity, especially in an electrolyte with a low methanol concentration (*e.g.*, ≤ 4%) under various applied potentials reported in the literature.^29,35,36^ However, formaldehyde, as a mid-stage oxidation product during methanol oxidation, has been largely overlooked, and the overoxidation of the formaldehyde to formic acid is therefore poorly understood. There have been rather limited details on the impact of applied potential or methanol concentration upon the selectivity of formaldehyde, which apparently possesses higher added value as the key commodity in chemical industries. Herein, we are able to demonstrate that the formaldehyde formation from methanol oxidation on NiOOH is dependent upon the methanol concentration and applied potential. The high selectivity of formaldehyde formation, however, requires a rather restrictive condition (low potential below the OER onset and high concentration of methanol). Outside the range of this electrocatalytic condition, the selectivity sharply decreases and results in an overoxidation pathway to continue oxidising the formaldehyde, which is, to the best of our knowledge, the first time reporting the conditions of high selectivity of formaldehyde formation from methanol oxidation. Despite the apparent conditions demonstrated herein, mechanistic studies of methanol oxidation to formaldehyde in the literature have been focused upon the structural transformation of the electrocatalyst during the catalysis based upon theoretical computations, which is insufficient to elucidate the diversion of selectivity in formaldehyde formation and the overoxidation of formaldehyde.^35,36^ The kinetics of oxidation charges, especially the evolution of these charges following the structural transformation, have still remained unclear. It is apparent that the applied potential and methanol (or water) concentration enabled different reaction pathways of methanol oxidation on NiOOH, which requires thorough kinetic investigation under these conditions, as we discuss below.

### Mechanistic distinction of methanol oxidation to formaldehyde and its overoxidation to formic acid

**Fig. 4:**
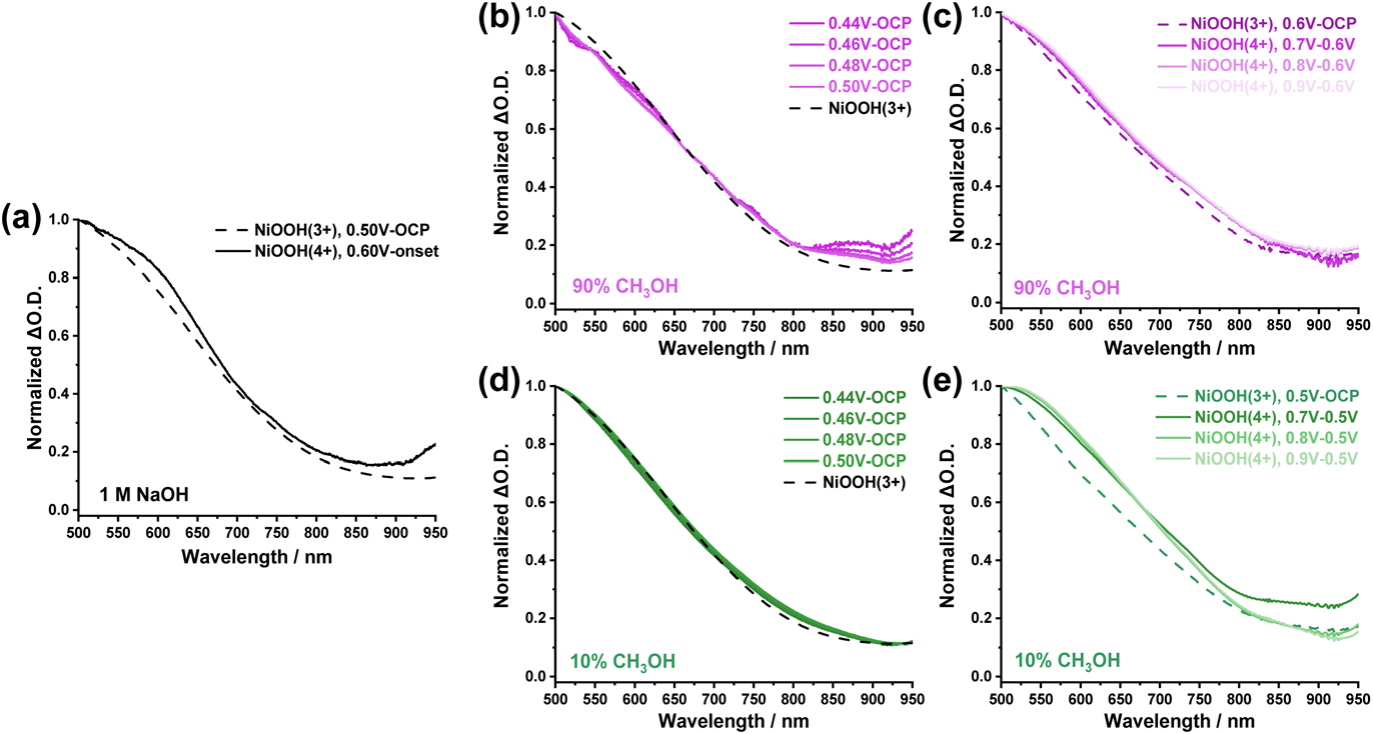
(a) Normalised SEC spectra of NiOOH(3+) (dashed line) and NiOOH(4+) (solid line) active species on the surface of NiOOH electrode during water oxidation (1 M NaOH). (b) Normalised SEC spectra of active species during the 90% methanol oxidation on NiOOH electrode at low potentials (<0.5 V_Ag/AgCl_). The ΔO.D. was obtained by subtracting the absorption at the open circuit potential (OCP). (c) Normalised SEC spectra of active species during the 90% methanol oxidation on NiOOH electrode at high potentials (>0.5 V_Ag/AgCl_). The ΔO.D. was obtained by subtracting the absorption at 0.6 V_Ag/AgCl_. (d) Normalised SEC spectra of active species during the 10% methanol oxidation on NiOOH electrode at low potentials (<0.5 V_Ag/AgCl_). The ΔO.D. was obtained by subtracting the absorption at OCP. (e) Normalised SEC spectra of active species during the 10% methanol oxidation on NiOOH electrode at high potentials (>0.5 V_Ag/AgCl_). The ΔO.D. was obtained by subtracting the absorption at 0.5 V_Ag/AgCl_.

We now turn to consider the mechanisms of methanol oxidation to formaldehyde and formic acid by employing the *operando* spectroelectrochemical (SEC) technique. Fig. 4a shows SEC spectra of NiOOH at 0.5 V_Ag/AgCl_*versus* the water oxidation potential at 0.6 V_Ag/AgCl_ in 1 M NaOH. A broad absorption was observed, which was attributed to the NiOOH(3+) generated in the region of the first redox peak shown in Fig. 1a, whilst there was no faradaic current of the OER taking place. When the applied potential was higher than the onset (0.5 V_Ag/AgCl_), a distinct red shift of the spectrum was observed due to the generation of NiOOH(4+), consistent with two species identified from the SEC results of NiOOH in our previous study, where the NiOOH(4+) was responsible for electrocatalytic OER.^21^ Detailed SEC spectra of NiOOH(3+) and NiOOH(4+) as a function of applied potential are provided in Fig. S9 in the SI. We also noticed that the SEC spectral shapes of NiOOH(3+) and NiOOH(4+) are rather similar, with a subtle broadening towards the higher wavelength region. Such broadening is likely due to the change in the d-orbital occupancy and d–d transition by removing one electron from NiOOH(3+) to allow the absorption of higher wavelength photons. Therefore, with this sensitive broadening phenomenon, we were able to carry out kinetic analyses to understand the reaction mechanism as discussed below.

We now correlate the SEC results of electrocatalytic methanol oxidation by NiOOH with the selectivity and Faraday efficiency determined above. In the presence of high concentration methanol (90%), the spectra at low potentials (<0.5 V_Ag/AgCl_) were also observed, as shown in Fig. 4b, which resembles the NiOOH(3+) SEC spectrum measured in 1 M NaOH, although there was no clear redox peak observed in the CV results in Fig. 1b. As the potential range for NiOOH(3+) generation also corresponds to the ∼100% Faraday efficiency for formaldehyde formation, it is then clear that the NiOOH(3+) is primarily the catalytic species responsible for methanol oxidation under high-selectivity conditions. This is also supported in Fig. S10, where both the optical density of NiOOH (3+) and the steady-state current were compared as a function of applied potential. In 1 M NaOH, the onset potential for NiOOH (3+) is around 0.34 V_Ag/AgCl_, whilst the onset potential for water oxidation is ∼0.48 V_Ag/AgCl_. The 0.14 V gap clearly indicates that the NiOOH(3+) is insufficient to drive water oxidation, possibly due to the lack of a thermodynamic driving force. In contrast, the generation of NiOOH(3+) in the 90% methanol electrolyte perfectly correlates with the steady state current as a function of applied potential, suggesting that these NiOOH(3+) species are active for the oxidation of methanol to formaldehyde according to the ∼100% Faraday efficiency shown in Fig. 2.

When the applied potential was increased, the SEC spectra started to show a broadening and red shift compared to the NiOOH(3+) SEC data shown in Fig. 4c, consistent with the NiOOH(4+) formation under higher applied potential in the region of the OER, as we^21^ and others^20,57^ previously reported. In addition to the deconvoluted spectra shown in Fig. 4, red shift and broadening were also observed in a continuous spectral evolution shown in Fig. S11, following the forward scan of applied potential in 1 M NaOH, 10% methanol, and 90% methanol electrolytes, clearly demonstrating that NiOOH oxidation state is modulated by applied potentials, which varies the reaction pathway and selectivity. Therefore, even in the presence of 90% methanol, the NiOOH(4+) was inevitable to form with higher energetics compared to NiOOH(3+). Therefore, the low Faraday efficiency for formaldehyde formation and overoxidation to formic acid under a strong applied potential is likely due to the higher oxidation power of NiOOH(4+). We are aware that other studies proposed a NiOOH mechanism under strong anodic potential, likely resulting from the NiOOH(4+) for formaldehyde formation using Raman spectroscopy.^30,36,60^ The Faraday efficiency and selectivity for formaldehyde formation, however, was either reported at a rather modest level (*e.g.* 20% of Faradaic efficiency),^37^ or unreported (only formic acid yield was reported).^29,35,36^ These observations are consistent with our SEC data that showed NiOOH(4+) to be too powerful to control the oxidation of methanol and formaldehyde. We also note that NiOOH(4+), generated under strong applied potential, provides sufficient oxidation power to drive OER in electrolytes without methanol.^20,21,57^ The strong oxidation power in NiOOH(4+) initiating the uncontrollable methanol oxidation is also supported by a recent study of NiOOH with a Ni(4+) phase in the bulk of NiOOH, far away from the Helmholtz double layer at the electrode–electrolyte interface, continuing to provide oxidation power to drive the surface NiOOH for OER.^61^ In parallel, in 10% methanol, similar NiOOH(3+) and NiOOH(4+) were observed in the SEC (Fig. 4d and e). However, one significant phenomenon is the low Faraday efficiency (4.3%) measured at 0.45 V_Ag/AgCl_ in 10% methanol (Fig. 2), which is in sharp contrast to the 90% methanol condition under the same applied potential (*i.e.*, ∼100% Faraday efficiency and selectivity). These results strongly indicate that another reaction pathway occurs during the interaction with water molecules, as discussed below. In terms of the oxidation power of NiOOH(4+), in the presence of methanol, the Faraday efficiency of OER was close to zero above 10% methanol, suggesting that NiOOH(4+) are more efficient to oxidise methanol than water molecules unless the methanol concentration decreased to below 10%, resulting in a more concentration-controlled kinetic competition between water molecules and methanol shown in Fig. S8. Nevertheless, the spectral analyses reported herein, for the first time, elucidate the operating requirement for the NiOOH active species (NiOOH(3+) before OER) and electrocatalytic cell conditions (>90% methanol concentration) for highly selective and efficient formaldehyde synthesis.

We now focus on the mechanism of ∼100% selectivity for formaldehyde formation by the NiOOH(3+). The aforementioned results clearly demonstrate that NiOOH(3+) is the active species responsible for selective formaldehyde formation. The condition has been confined within a limited water concentration to avoid overoxidation of formaldehyde to formic acid. Therefore, there is clearly a water-controlled kinetics in methanol oxidation by NiOOH(3+). We employed voltage-induced absorption (VIA) spectroscopy, previously employed to study electrocatalytic water splitting using metal oxides,^42^ to quantitatively analyse the methanol oxidation kinetics by NiOOH(3+) as a function of water concentration. A pulsed step potential (10 s on/20 s off) between the OCP and the applied potential in the NiOOH(3+) region was applied to the NiOOH electrode, and the corresponding pulsed optical absorption response was simultaneously recorded. The applied potentials were controlled between 0.4 and 0.5 V_Ag/AgCl_ to ensure the generation of NiOOH(3+) before the potential of NiOOH(4+). Quasi-steady state VIA data and methanol oxidation current were recorded at 10 s, as shown in Fig. S13. A rate law analysis was employed to investigate the reaction kinetics (eqn (1)); the concentration of NiOOH(3+) species was determined *via* calculation of the extinction coefficient. The effect of water content on the oxidation mechanism of methanol was investigated using a rate law previously developed for electrocatalytic water oxidation.^20,21^ Indeed, the rate law analysis applies to the population model, which has been reported in literature for electrodes with discrete semiconductor energy states in both photoelectrochemical and electrochemical reactions.^62^ In this model, the energy that drives redox reactions remains constant with applied potential. It is considered to be a process of accumulation of active species. Based on this, we can investigate a kinetic study of methanol oxidation by the single active species NiOOH(3+).

1 log *J* = log *k* + *α* log[NiOOH(3+)]

where *J* (e^−1^ s^−1^ nm^−2^) is the reaction rate of the water oxidation; *k* is the rate constant; *α* is the order of reaction, and [NiOOH(3+)] (nm^−2^) corresponds to the surface density of the accumulated NiOOH(3+) species on the NiOOH electrode. The extinction coefficients of 3260 M^−1^ cm^−1^ for water oxidation and 3250 M^−1^ cm^−1^ for 90% methanol oxidation (Fig. S12) indicate that the extinction coefficient of NiOOH(3+) is independent of the methanol concentration.

**Fig. 5:**
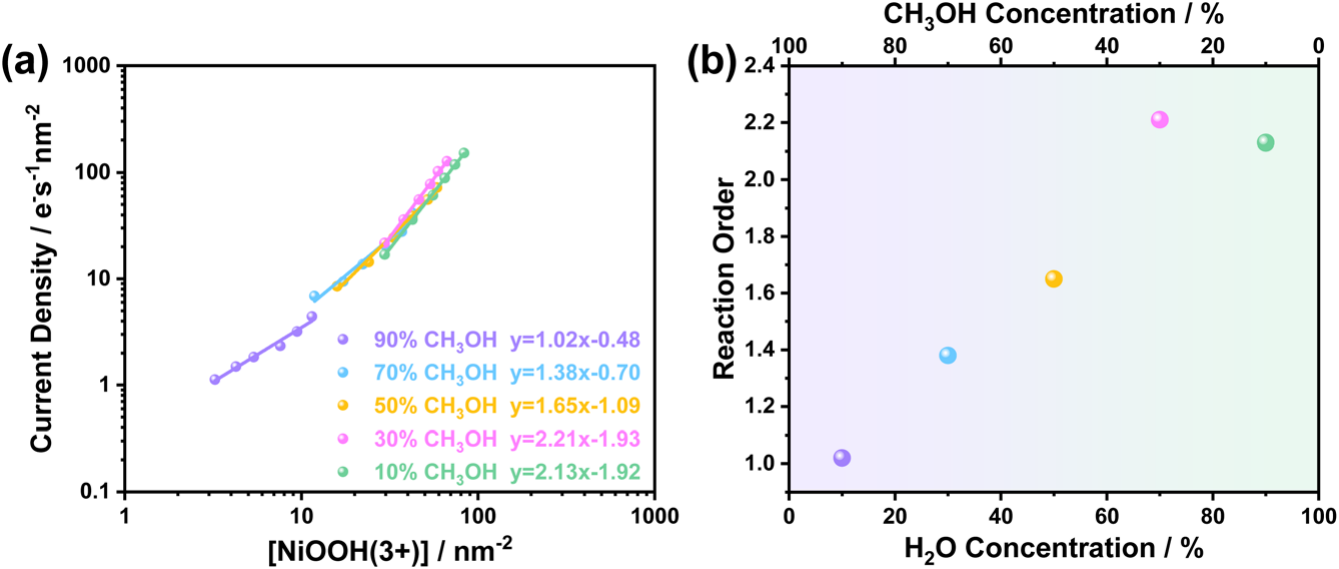
(a) Rate law analyses of methanol oxidation current density *versus* the surface concentration of NiOOH(3+). (b) Changes in the methanol oxidation reaction order as a function of water (or methanol) concentration.

Fig. 5a shows the rate law analyses of NiOOH(3+) as a function of methanol concentration in the electrolyte. First-order kinetics were observed when there is 90% methanol (or 10% H_2_O) in the electrolyte. As the selectivity and Faraday efficiency have been determined to be ∼100% for formaldehyde formation, the first-order kinetics then correspond to a single oxidation process by NiOOH(3+) at the rate-limiting step (RLS) of formaldehyde formation. In contrast, it is striking that the reaction order in NiOOH(3+) increased from 1 to 2 with a decrease in the methanol concentration to 10%, as summarised in Fig. 5b. The reaction order as a function of methanol or H_2_O concentration is in perfect correlation with the Faraday efficiency for formaldehyde formation as a function of methanol concentration in Fig. 2. Additionally, from the CV and steady-state current data in Fig. 1, we also observed an increase in catalytic current corresponding to the decrease in methanol concentration, indicating faster reaction kinetics. However, in contrast to the increasing trend in CV with increasing water content, such higher reaction kinetics did not result in an increased selectivity of formaldehyde formation. Rather, decreased Faraday efficiency and formic acid yield were observed according to the NMR results in Fig. 3. At the low methanol concentration, the oxidation product was formic acid, as confirmed by ^1^H NMR (Fig. 3). The reaction order gradually shifted to the 2nd order suggesting that the oxidation pathway was greatly changed by H_2_O from methanol oxidation *via* first-order of formaldehyde formation to the second order of formic acid formation in NiOOH(3+), where the intermediate formaldehyde was further activated in the presence of H_2_O, although no redox activity of H_2_O involved in the overoxidation of formaldehyde as discussed below. We also note that the presence of water changes the course of the methanol oxidation pathway. An increase in water concentration is more favorable to enhance interactions with C–H bonds and provide –OH groups for overoxidation to form formic acid.

**Fig. 6:**
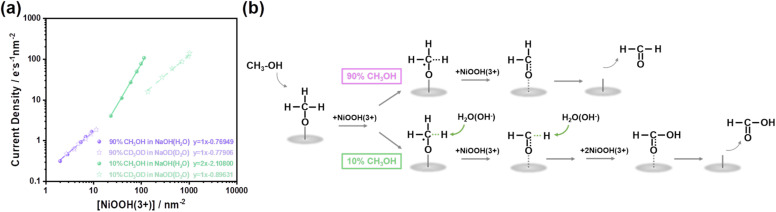
(a) Rate law analyses of 90% CH_3_OH (purple, sphere), 90% CD_3_OD (purple, star), 10% CH_3_OH (green, sphere), and 10% CD_3_OD (green, star) oxidation. (b) Schematic representation of methanol oxidation reaction pathways.

We now consider a possible mechanism of methanol oxidation by NiOOH(3+) to formaldehyde and overoxidation to formic acid according to the product determined above. Fig. 6a compares the rate law results by NiOOH(3+) in 10% and 90% methanol in Fig. 5a, corresponding to formic acid and formaldehyde, respectively. In 90% methanol, the first-order kinetics suggest a single oxidation by NiOOH(3+) on the surface-adsorbed methanol. As summarised in Fig. 6b, single oxidation yields a methoxide radical, supported by an identical first-order rate constant measured in H/D isotope exchange of methanol, NaOH, and H_2_O, as shown in Fig. 6a and S14a, corresponding to a kinetic isotope effect (KIE = *k*_H_/*k*_D_) equal to 1. The result of KIE = 1 further indicated that C–H bond cleavage does not limit first-order oxidation to methanol. After the initial C–H bond cleavage, further C–H activation and cleavage appear to be limited, resulting in the oxidation being terminated at the formaldehyde stage.

Conversely, in the 10% methanol electrolyte, where H_2_O occupied the majority of species, not only did H_2_O alter the oxidation product, but also the reaction kinetics were greatly changed from the first order (∼100% formaldehyde) to the second order (overoxidation). As the oxidation of methanol requires breaking the C–H bond once for formaldehyde and twice for formic acid, H_2_O is clearly involved in the activation of the second C–H bond and the addition of the –OH group to the methanol carbon to form formic acid. In addition, the presence of H_2_O clearly assisted with the activation of C–H bonds in methanol, likely to lower the energetics of C–H bonds, similar to the photo-oxidation of alcohol molecules by TiO_2_ semiconductor, as we reported previously.^43^ Therefore, methanol oxidation tends to be a process of sequential oxidation at a single active site, without involving synergistic interactions between sites on the NiOOH surface. In this work, particular attention should be paid to the activation effect of water molecules on reaction intermediates. Concurrently, it is crucial to ensure the formation of the NiOOH(3+) active species on the electrode surface, a point that has been discussed in detail in Fig. 4 above. In addition to the reaction, the impact of H_2_O upon the formaldehyde selectivity is also shown as the change in the open circuit potential in Fig. S15. The OCP was relatively constant as a function of methanol oxidation at approximately −0.4 V_Ag/AgCl_ until 70%, and a significant increase occurred when the concentration reached at 90%. The change in OCP is likely related to the methanol adsorption on the NiOOH surface in kinetic competition with water molecules. This trend is also in perfect agreement with the Faraday efficiency shown in Fig. 2, where overoxidation occurred with water molecules adjacent to the catalytic sites. In 90% methanol, the catalytic sites were most likely to be surrounded by methanol rather than water, thus screening water molecules away from the catalytic site, directly resulting in ∼100% Faraday efficiency and selectivity of formaldehyde formation. We are aware that a detailed investigation of this overoxidation with C–H bond activation requires individual oxidation of formaldehyde alone. However, given the fact that a polymerization process of formaldehyde is intended to occur in a pure formaldehyde solution, a high concentration (∼10%) of methanol is often used to stabilise formaldehyde, making the individual oxidation of formaldehyde practically impossible. In addition, the bonding site between the formaldehyde and the NiOOH surface is likely to change for formaldehyde re-adsorption onto the NiOOH surface. Despite these challenges, our spectroelectrochemical and kinetic assays are able to provide a fresh perspective on the oxidation of methanol to formaldehyde and formic acid in a continuous step-by-step motion of structural transformation.

The impact of C–H bond strength upon the selectivity during methanol oxidation is now discussed. Fig. 6a also shows the rate law analyses of H/D exchanged methanol oxidation in 90% and 10% concentration. In contrast to the first order and KIE = 1 in 90% methanol-*d*_4_, the reaction order shifted from the first order in 10% methanol-*d*_4_ compared to the second order measured in 10% methanol. This reaction order change, simply by the deuterium exchange, suggests that the condition of C–H activation has been greatly affected by the bond strength of C–H/C–D. Additionally, comparing with methanol-*d*_4_/H_2_O (NaOH) and methanol-*d*_4_/D_2_O (NaOD), the rate law analyses exhibited an identical reaction order (first order) and rate constant (∼0.17 s^−1^ in 90% methanol-*d*_4_ verse ∼0.13 s^−1^ in 10% methanol-d_4_), strongly suggesting that C–D activation in the methanol-*d*_4_ was independent of water molecules (Fig. S14) and the H/D exchange at the O–H bond cleavage in the methanol hydroxyl group did not kinetically limit the overall reaction. Based on this, it is further clarified that the adsorption process of methanol molecules and water molecules (involving O–H bond cleavage) does not affect the reaction kinetics of methanol oxidation. More importantly, whether the C–H bond cleavage of methanol molecules is influenced by water molecules determines the selectivity of the products. Despite the same first-order kinetics, the Faraday efficiency of formaldehyde-d_2_ formation was rather different, where ∼100% Faraday efficiency was measured in 90% methanol-*d*_4_ compared to a < 20% Faraday efficiency in 10% methanol-*d*_4_ (Fig. S16). The difference in the methanol concentration-dependent Faraday efficiency clearly demonstrates the hidden role of the water molecule in the C–H (C–D) activation and the reaction selectivity of methanol oxidation. Finally, it is striking that the C–H bond strength changed the reaction pathway and selectivity, which has not been considered in the electrocatalytic and photoelectrocatalytic system for methanol oxidation to formaldehyde, using NiOOH or NiO_*x*_ derivatives as the electrocatalyst or co-catalyst in photoelectrocatalysis.^57,63^ It is also noted that the NiOOH electrode on FTO investigated herein is underoptimised in performance due to the requirement for optical transmission. The optimization of NiOOH thickness and the use of more conductive substrates significantly improve the overall rate of formaldehyde synthesis. In addition, despite the limited potential window allowed for selective formaldehyde formation, nanostructuring of the NiOOH surface is strongly recommended to increase the quantity of catalytic sites, which will eventually increase the overall current amplitude for selective formaldehyde formation. As 90% methanol concentration is critical for the 100% formaldehyde formation, we propose that modifications of the electrocatalytic system are required towards an online and full-time monitoring system with a continuous flow configuration to enable the concentration to be fixed at above 90%. Such a configuration in device engineering is equally critical for the large-scale production of formaldehyde using the electrocatalytic approach. In addition, a recent study of electrochemical alcohol oxidation employed bicarbonate to salt out the aldehyde, which effectively mitigated the mass transfer limitations caused by the accumulation of aldehyde around the electrode surface.^37^ These reported materials and system engineering clearly demonstrate the promise of combining the fundamental understanding of the reaction and the engineering of the system for efficient green production of aldehyde *via* electrochemical oxidation of alcohols. Nevertheless, our results presented herein not only discover the unnoticed condition for selective value-added formaldehyde synthesis but also strongly emphasise the consideration of general C–H bond strength upon its activation towards a manipulatable oxidation of hydroxyl group for both value-added synthesis, such as glycerol oxidation and alcohol fuel cells, as an efficient chemical-to-electricity system.

## Conclusions

In this study, we demonstrate that NiOOH enables ∼100% selectivity for formaldehyde formation during electrocatalytic methanol oxidation. High methanol concentration and applied potentials near the current onset are two key factors that result in ∼100% selectivity and Faraday efficiency. Spectroelectrochemistry is employed to elucidate how formaldehyde and overoxidation to formic acid take place during methanol oxidation under different electrochemical conditions. Our spectroscopic assay confirms the reaction pathways of formaldehyde formation and its overoxidation, and elucidates that C–H bond activation appears to be the key determinant for the selectivity of formaldehyde formation and the overoxidation, driven by either water molecules or applied potentials. NiOOH is then considered a promising electrocatalyst for methanol oxidation to higher-value formaldehyde, offering a low-cost, greener, and safer synthetic approach for industrial-scale production.

## Author contributions

Z. M. and Y. M. conceived the project. Z. M. performed the measurements and analysed the data. Y. Y. prepared the electrode samples. Z. L. contributed to the Raman measurements. Z. M. and Y. M. co-wrote the manuscript with contributions from all authors.

## Conflicts of interest

There are no conflicts to declare.

## Supplementary Material

SC-OLF-D6SC00905K-s001

## Data Availability

The data supporting this article are included in the supplementary information (SI). Supplementary information: materials preparation, characterisation methods (SEM, XRD, XPS, Raman, electrochemical characterisations; Faraday efficiency of formaldehyde formation; NMR; O_2_ and H_2_ quantification; SEC and VIAS data). See DOI: https://doi.org/10.1039/d6sc00905k.
