# Supplementary material for: Unraveling the hidden conditions in NiOOH for electrocatalytic oxidation of methanol to formaldehyde with unity Faraday efficiency and selectivity
Source: Chem Sci. 2026 Mar 23;17(20):9960–70. doi: 10.1039/d6sc00905k (PMC13055912; doi:10.1039/d6sc00905k)
Supplement: SC-017-D6SC00905K-s001 [file SC-017-D6SC00905K-s001.pdf]

Supplementary Information (SI) for Chemical Science.
This journal is © The Royal Society of Chemistry 2026

Supplementary Information for

Unraveling The Hidden Conditions in NiOOH for Electrocatalytic Oxidation of Methanol to Formaldehyde with Unity Faraday Efficiency and Selectivity

Zixuan Ma,[a] *Yuling Yuan,*[a] Ze Lv,[a] *and Yimeng Ma**[a]

College of Chemistry and Chemical Engineering, Donghua University, Shanghai, 201620, China

AUTHOR INFORMATION

Corresponding Author

*Correspondence: yimeng.ma@dhu.edu.cn

1. Experimental section

1.1 Materials

Fluorine-doped tin oxide conductive glass (FTO, sheet resistance: < 15 ohm sq^{-1}, transmittance: > 83%, thickness: 2.2 mm) was purchased from Zhuhai Kaivo Optoelectronic Technology Co., Ltd. Nickel (II) sulfate hexahydrate ($NiSO_4 \cdot 6H_2O$, ≥ 98.5%, AR), hydrochloride (HCl, 36.0 ~ 38.0%, AR), Sodium hydroxide (NaOH, ≥ 96.0%, AR), and Methanol (CH_3OH, ≥ 99.7%, GR) were all purchased from Sinopharm Chemical Reagent Co., Ltd. Sodium deuteroxide solution (NaOD, 99.5% D, basicity 30%), Deuterium oxide (D_2O, 99.9% D), and deuterated methanol (CD_3OD, 99.8%D) were purchased from Shanghai Haohong Scientific Co., Ltd. 4-amino-3-hydrazino-5-mercapto-1,2,4-triazole (AHMT, 99%, AR) was purchased from Shanghai Rhawn Chemical Technology Co., Ltd. Formaldehyde (HCHO, 37.0 ~ 40.0%, AR) was purchased from Shanghai Titan Technology Co., Ltd. The resistivity of deionised water used in the experiment was 18.25 MΩ cm at 298 K.

1.2 Electrodeposition of NiOOH electrodes

Nickel oxide hydroxide (NiOOH) electrode was prepared using the electrodeposition method as previously reported.[1, 2] Prior to electrodeposition, Fluorine-doped tin oxide conductive glass (FTO) required pretreatment.[3] The substrates were first cut into 1.5 cm × 2 cm pieces, then ultrasonically cleaned sequentially with detergent, deionised water, and ethanol for 30 minutes each. Finally, the FTO was placed in a crucible and calcined in a muffle furnace (LE 6/11/R7, Nabertherm) at 500°C for 2 h to completely remove surface contaminants.

NiOOH was electrodeposited onto FTO substrates (deposition area: 1.5 cm × 1.5 cm) using a CHI 1140C potentiostat (CH Instruments). The cleaned FTO, nickel foam, and Ag/AgCl electrode (saturated KCl solution) were served as the working electrode (WE), counter electrode (CE), and

reference electrode (RE), respectively. The deposition was performed at 1.2 $V_{Ag/AgCl}$ in a 100 mM $NiSO_4 \cdot 6H_2O$ aqueous solution (pH adjusted to ~ 7.0) with a water bath maintained at 75°C, until a total charge density of 0.15 C cm^{-2} accumulated on the FTO surface. Subsequently, the prepared NiOOH samples were rinsed with deionised water and air-dried overnight. For the deposition of NiOOH on a carbon paper (TGP-H-060, Suzhou Sinero), the identical method and conditions were applied, yielding a similar amorphous structure of NiOOH as confirmed by structural analysis discussed below.

1.3 Characterizations of NiOOH electrodes

The surface morphology and cross section of NiOOH electrodes were characterised using field emission scanning electron microscopy (FE-SEM, SU8010, Hitachi). The crystalline phase was analysed by X-ray diffraction (XRD, Bruker D8 Advance) with diffraction patterns scanned from 10° to 80° in 2θ. Chemical states and coordination environments were probed via X-ray photoelectron spectroscopy (XPS, Escalab 250Xi), where all spectral were calibrated against the C 1s peak at 284.8 eV binding energy. Raman spectra were performed using a Thermo Scientific DXR2xi Raman imaging microscope (Thermo Fisher Scientific, Madison, Wis., USA) equipped with a 100× lense under 532 nm laser excitation.

1.4 Electrochemical characterizations of NiOOH electrodes

Electrochemical testing was performed using a standard three-electrode configuration, with the NiOOH electrode as the working electrode, platinum mesh (2 cm × 2 cm) as the counter electrode, and an Ag/AgCl electrode (saturated KCl) as the reference electrode. Electrochemical characterization of NiOOH electrode was carried out using an Autolab potentiostat (PGSTAT204, Metrohm). Cyclic voltammetry (CV) at a scan rate of 10 mV s^{-1} and chronoamperometry (CA)

with steady-state current acquisition at specified applied potentials were conducted in methanol from 0% to 90% in 1 M NaOH (methanol: water, v/v%).

1.5 Faraday efficiency of methanol oxidation to formaldehyde

The AHMT spectrophotometric method was employed for quantitative determination of formaldehyde generated via electrocatalytic oxidation of methanol by NiOOH electrode. Formaldehyde condensed with AHMT reagent under alkaline conditions, followed by aerial oxidation to form a purple-red compound, 6-mercapto-s-triazolo [4, 3-6]-s-tetrazine. The solution absorbance exhibited linear proportionality to formaldehyde concentration and was quantified at 549 nm using UV-vis spectroscopy. The AHMT spectrophotometric method was modified in this study relative to previous reports.[3-6]

Standard curve determination: Formaldehyde solutions (0 - 15 ppm, 10 mL) were mixed with 5 M NaOH (5 mL) and 0.5wt% AHMT solution (10 mL). After 30 min agitation for chromogenic reaction, absorbance values at 549 nm were measured for each formaldehyde concentration, followed by construction of the calibration curve.

Formaldehyde Faraday efficiency (FE) determination: Steady-state current recorded during methanol electrocatalytic oxidation by NiOOH at applied potential enabled calculation of the theoretical formaldehyde yield. Subsequently, the post-reaction solution (10 mL) was mixed with 5 M NaOH (5 mL) and 0.5wt% AHMT solution (10 mL) for chromogenic development (30 min). Absorbance at 549 nm was then measured and interpolated into the pre-established calibration curve to quantify actual formaldehyde production.

The formaldehyde Faraday efficiency can be calculated using the following Equation S1:

$$FE(\%) = \frac{n_{detected\ HCHO}}{n_{theoretical\ HCHO}} \times 100\% \quad (S1)$$

where $n_{detected\ HCHO}$ is mol of detected HCHO, $n_{theoretical\ HCHO}$ is mol of theoretical calculated HCHO from electrochemical measurement.

1.6 Analysis of formic acid in the products of methanol oxidation reaction

Nuclear Magnetic Resonance (NMR, Avance III HD 600 MHz, Bruker) was used to analyse the products of the methanol oxidation reaction catalysed by NiOOH electrode. The reaction systems with 10% methanol (in 1 M NaOH electrolyte) and 90% methanol (in 1 M NaOH electrolyte) were oxidised at applied potentials of 0.45 $V_{Ag/AgCl}$ and 0.75 $V_{Ag/AgCl}$ for 54 h, respectively. Subsequently, 500 μL of the post-reaction electrolyte was mixed with 500 μL of D_2O.[7] The resulting solution was transferred to an NMR tube, thoroughly mixed, and subjected to ^1H NMR spectroscopic analysis.

1.7 O_2 and H_2 quantification during electrochemical methanol oxidation

An automated all-glass gas circulation system (LABSOLAR-6A, PerfectLight) coupled with gas chromatography (GC 9790Ⅱ, Fuli Instruments) was employed to monitor H_2 and O_2 evolution during the electrocatalytic oxidation of x% methanol on NiOOH electrode. A three-electrode configuration utilised NiOOH/FTO as the working electrode, platinum mesh (1 cm × 1 cm) as the counter electrode, and Ag/AgCl electrode (saturated KCl solution) as the reference electrode. A constant potential of 0.75 $V_{Ag/AgCl}$ was applied using CHI 1140C potentiostat (CH Instruments) to record reaction current. It should be noted that, the gas test system was under a near-vacuum environment at ~1.5 kPa in order to allow dissolved gas products to diffuse from the electrolyte into the gas circulation and accumulation system. Therefore, limited by the sensitivity of the

instrument and disturbances to the gas circulation system caused by the decreased boiling point of the methanol-water mixture under vacuum, long-term gas monitoring data may exhibit certain fluctuations. However, this does not affect the subsequent quantitative analysis of oxygen by Faraday efficiency being generated in the presence of methanol. Throughout the reaction, 0.6 mL gaseous products were sampled by GC every 30 min, separated via a 5A column (RubyBondTM) under argon carrier gas, and detected by thermal conductivity detector (TCD) at 398 K. Concentrations of H_2 and O_2 were determined using standard calibration curves, with FE calculated by Equation S2:

$$FE = \frac{\text{charges for evolution}}{\text{total charges generated}} = \frac{n \times z \times F}{I \times t} \times 100\% \qquad (S2)$$

where n (mol) is the moles of oxygen or hydrogen generated; I (A) is the current generated during the electrochemical oxidation; t (s) is the electrochemical measurement time; z is the number of electrons involved in the oxygen evolution reaction (OER, z = 4) and hydrogen evolution reaction (HER, z = 2); F ($C\ mol^{-1}$) is the Faraday constant (96485 $C\ mol^{-1}$).

1.8 Operando spectroelectrochemistry

The operando spectroelectrochemical (SEC) measurement was performed to monitor optical absorption spectral changes of the NiOOH electrode surface at each applied potential. The electrode was placed in a three-electrode quartz cell. A 150 W tungsten lamp provided probe light source, which passed through a monochromator (TLS15-T150A, Zolix Instruments). The optical signal was recorded using a high-sensitivity spectrometer (QE65Pro, Ocean Optics). Corrtest potentiostat (CS150M) was used to apply open circuit potential (OCP) for 30 min on the NiOOH electrode to obtain stable electrochemical spectra. Then the current was stabilised for 150 s at each

potential. Subsequently, 30 spectra were recorded every 3 seconds and averaged to obtain absorption spectra by subtracting the reference spectrum measured at OCP or at a specific potential.

1.9 Voltage-induced absorption spectroscopy

The voltage-induced absorption spectroscopy (VIAS) measurement was modified from the previous report.[8] The 10 s on 20 s off pulse was controlled by step potential measurement using a Corrtest potentiostat to obtain transient current data. Step potential was applied to the NiOOH electrode from OCP to each catalytic potential. The probe light was generated via the W lamp used for spectroelectrochemical measurements and filtered through long-pass filters (Hengyang Optics) and a bandpass filter (Rayan Optics) at the specified probe wavelength and then recorded by a homemade detector (Hamamatsu S5973). The optical data was analysed by a data acquisition device (USB-6361, National Instruments) to receive the voltage-induced absorption signal (ΔO.D.).

1.10 Extinction coefficient estimation

The extinction coefficient (ε ($M^{-1}cm^{-1}$)) of NiOOH(3+) reactive species on the surface of NiOOH electrode was quantified via step-potential spectroelectrochemistry. Consistent with the Lambert-Beer law (Equation S3), the optical absorbance (O.D.) demonstrates a linear correlation with the surface density of oxidation species at a particular wavelength,

$$\Delta \mathrm{O.D.} = \varepsilon \times \Delta \mathrm{C} \tag{S3}$$

where ΔO.D.is the change of the absorbance at a particular wavelength, ε ($M^{-1}cm^{-1}$) is the extinction coefficient and ΔC (M cm) is the change of the surface density of oxidation species.

The extinction coefficient of NiOOH(3+) species at the NiOOH electrode surface was measured in both water oxidation and 90% methanol oxidation systems, with the Corrtest potentiostat (CS150M) applying step voltages from the OCP to potentials generating NiOOH(3+), while simultaneously recording steady-state spectral data. At each catalytic potential, an oxidative current transient emerged before stabilizing. Upon switching the potential back to OCP, a reductive current transient occurred until current decayed to zero. By integrating this reductive transient, the surface density of the NiOOH(3+) species, C (M cm), involved in the reduction could be obtained. The absorbance (O.D.) at 500 nm was extracted from spectra data and combined with the Beer-Lambert law (Equation S3), the extinction coefficients, ε, can be derived from the slope value by plotting ΔO.D. (O.D. - $O.D._{OCP}$) against ΔC.

2. Supplementary Figures

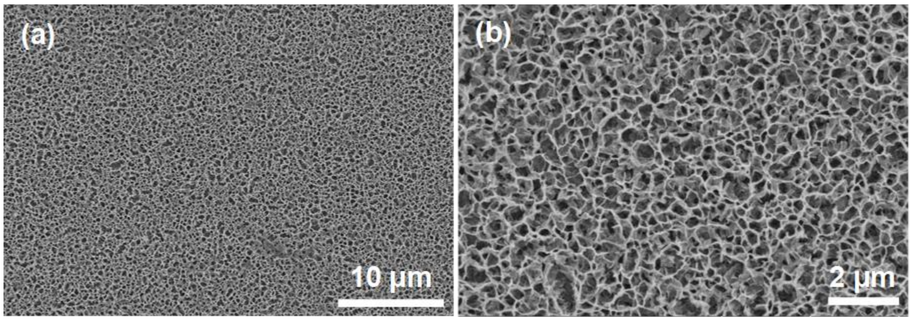

Figure S1. (a, b) SEM images of top view of NiOOH with different magnifications.

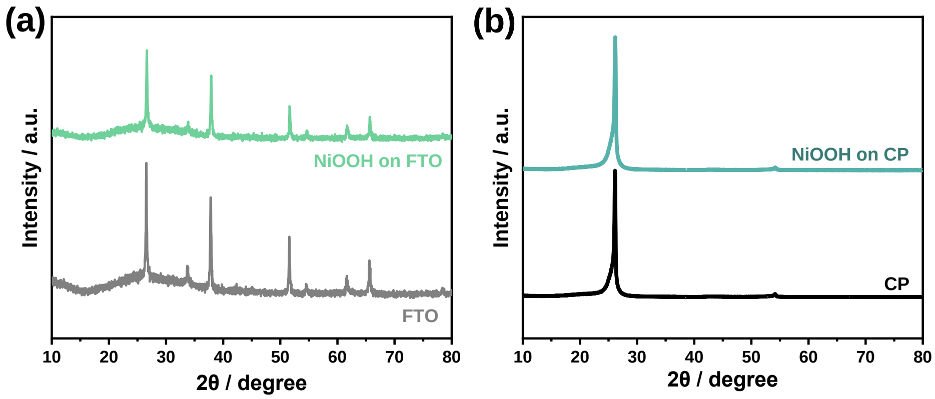

Figure S2. XRD characterizations of (a) NiOOH on FTO substrate (deposited charge density: 0.15 C cm^{-2}) and (b) NiOOH on carbon paper (CP) substrate (deposited charge density: 1.5 C cm^{-2}).

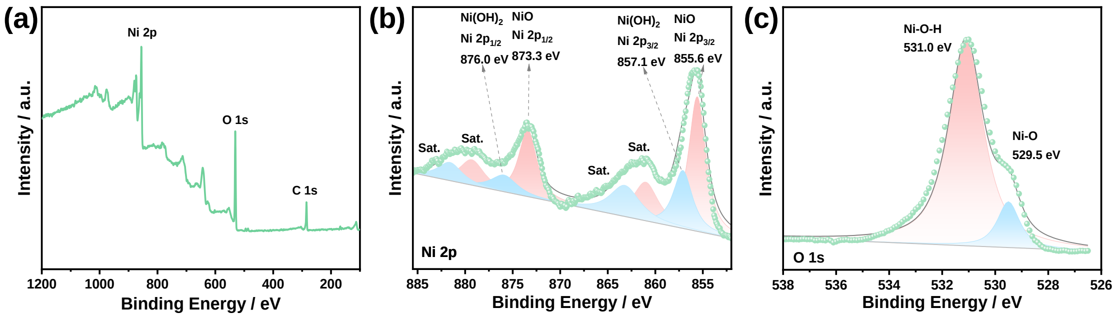

Figure S3. (a) the full survey XPS measurement data; (b) Ni 2p and (c) O 1s spectrum of NiOOH electrode as-prepared.

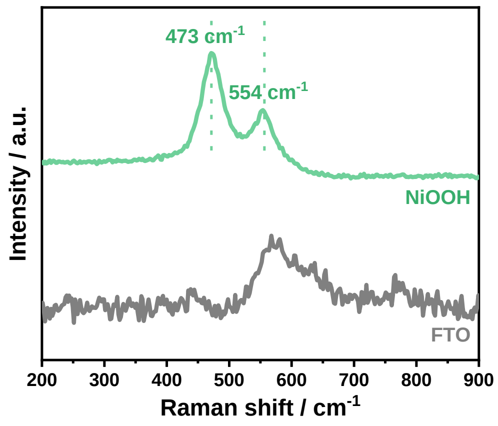

Figure S4. Raman spectroscopic results of NiOOH (green line) and FTO (grey line).

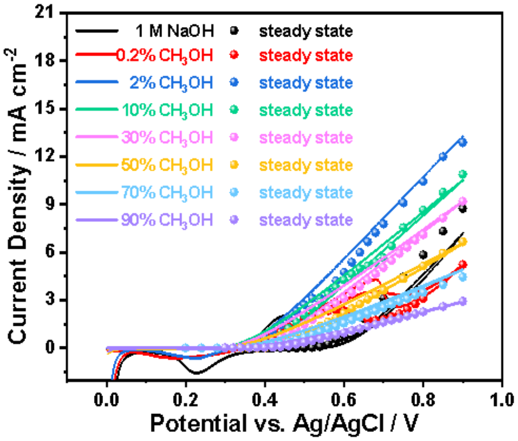

Figure S5. Cyclic voltammetry curves of oxygen evolution reaction and methanol oxidation reaction on NiOOH electrode and the corresponding steady-state current density (sphere). Scan rate: 10 mV s^{-1}.

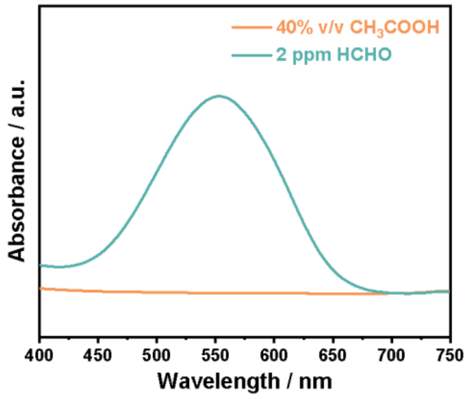

Figure S6. UV-vis absorption spectra of 4-amino-3-hydrazino-5-mercapto-1,2,4-triazole with 2 ppm formaldehyde and 40% v/v formic acid.

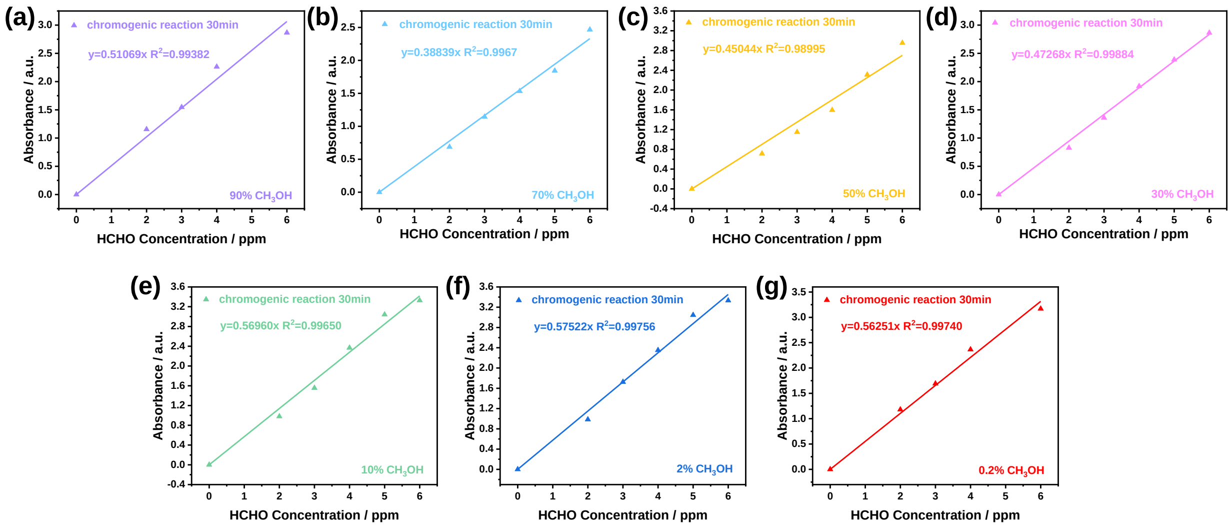

Figure S7. Standard curve establishment for formaldehyde in (a) 90% CH_3OH, (b) 70% CH_3OH, (c) 50% CH_3OH, (d) 30% CH_3OH, (e) 10% CH_3OH, (f) 2% CH_3OH, (g) 0.2% CH_3OH.

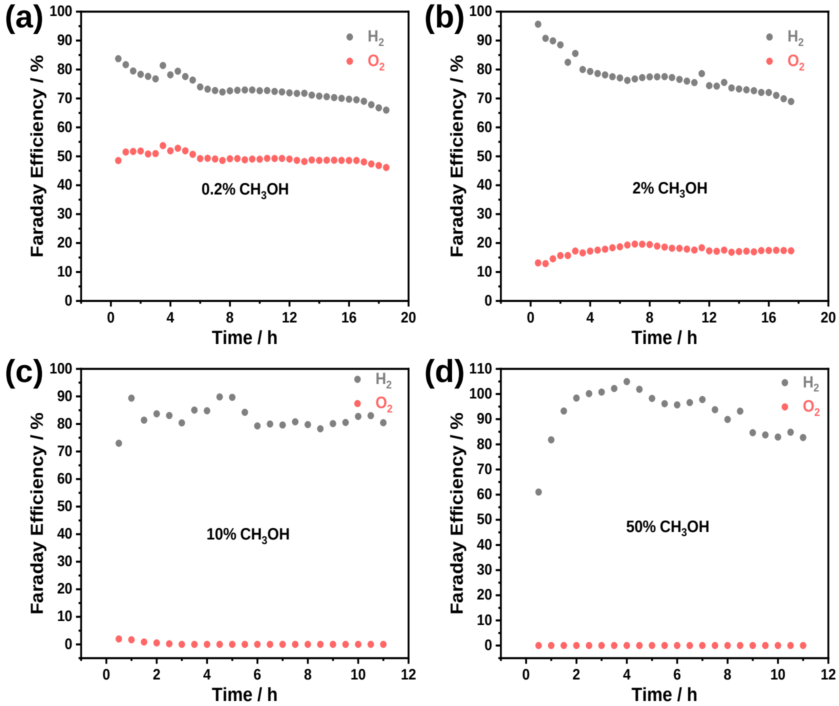

Figure S8. Faraday efficiency of H_2 and O_2 in (a) 0.2% CH_3OH electrochemical oxidation reaction, (b) 2% CH_3OH electrochemical oxidation reaction, (c) 10% CH_3OH electrochemical oxidation reaction, (d) 50% CH_3OH electrochemical oxidation reaction. Applied potential: 0.75 $V_{Ag/AgCl}$.

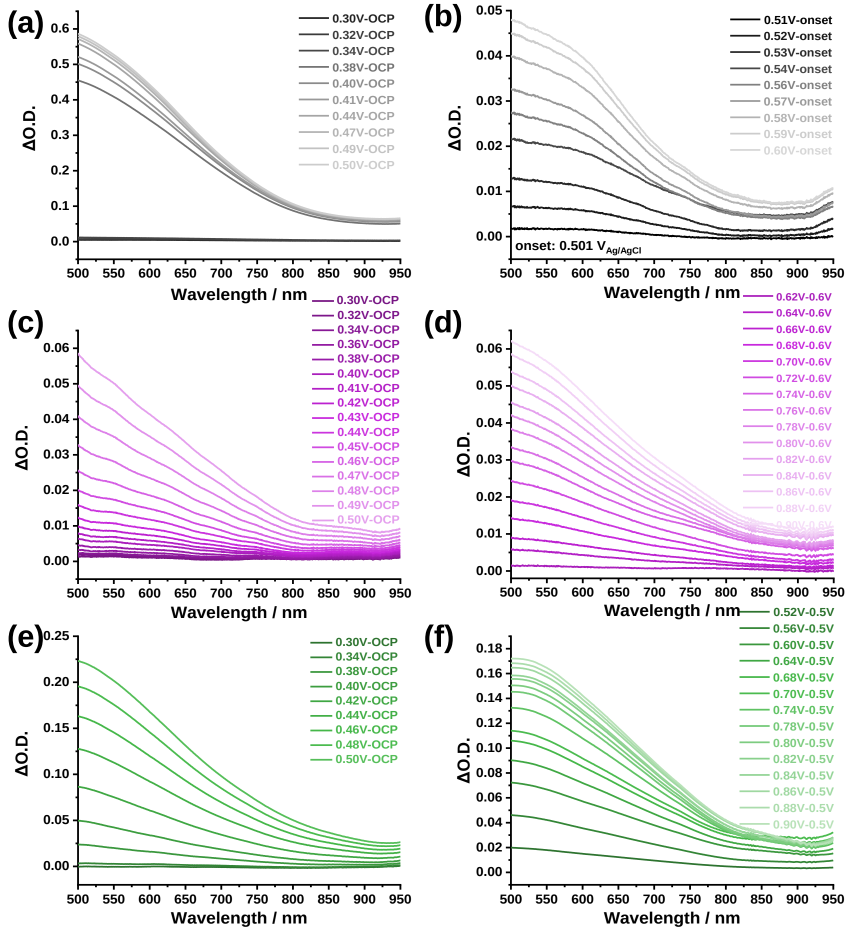

Figure S9. SEC spectra of NiOOH(3+) and NiOOH(4+) species on NiOOH electrode at different potentials. SEC spectra for electrocatalytic oxidation in 1 M NaOH: (a) ΔO.D. was obtained by subtracting the absorption spectrum at OCP. (b) ΔO.D. was obtained by subtracting the absorption spectrum at water-oxidation onset potential (0.5 $V_{Ag/AgCl}$). SEC spectra for electrocatalytic oxidation in 90% CH_3OH: (c) ΔO.D. was obtained by subtracting the absorption spectrum at OCP. (d) ΔO.D. was obtained by subtracting the absorption spectrum at 0.6 $V_{Ag/AgCl}$. SEC spectra for electrocatalytic oxidation in 10% CH_3OH: (e) ΔO.D. was obtained by subtracting the absorption spectrum at OCP. (f) ΔO.D. was obtained by subtracting the absorption spectrum at 0.5 $V_{Ag/AgCl}$.

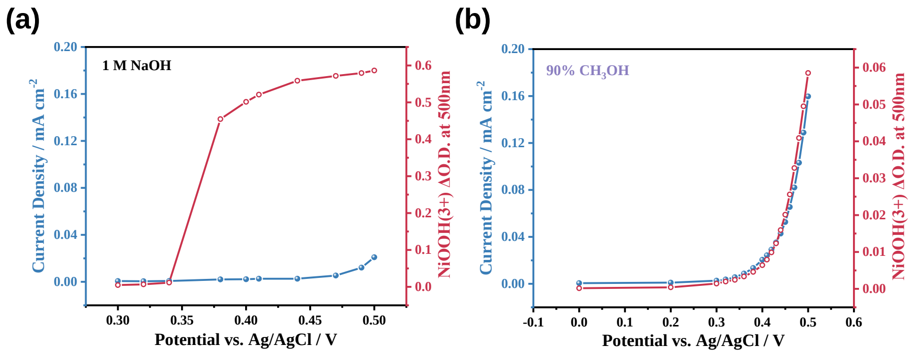

Figure S10. Correlation of the steady-state SEC amplitude of NiOOH(3+) at 500 nm with electrochemical current as a function of applied potential. (a) in 1 M NaOH and (b) in 90 % methanol electrolyte.

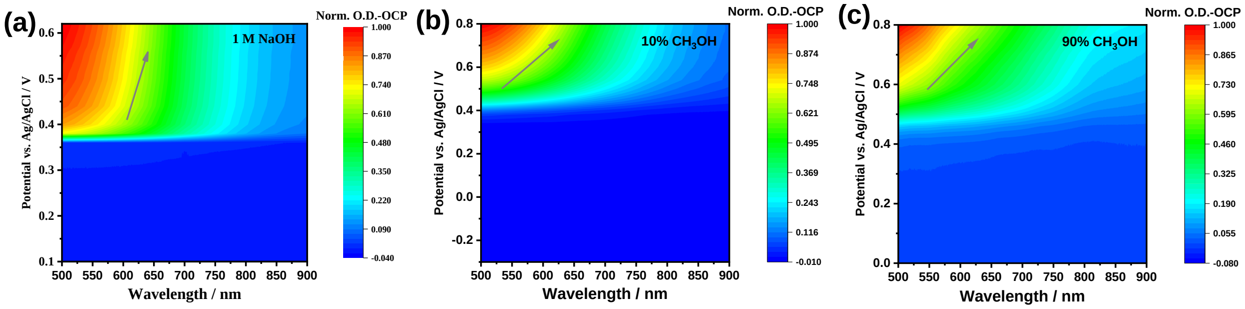

Figure S11. SEC spectra of NiOOH electrode following the forward scan of the applied potential measured in (a) 1 M NaOH, (b) 10 % methanol and (c) 90 % methanol. The spectra were recorded by subtracting their individual baseline spectrum at the open circuit potential.

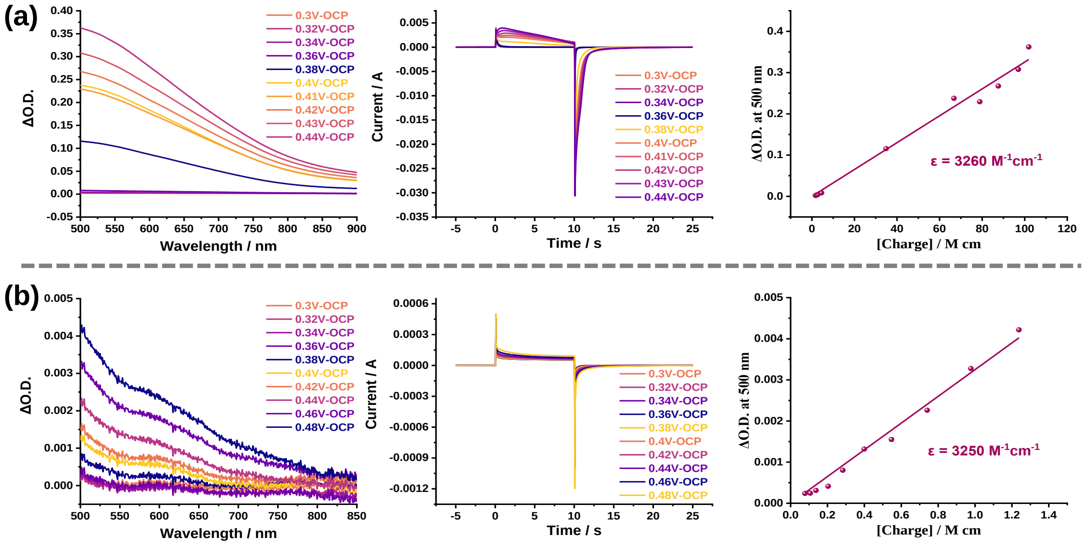

Figure S12. The absorption data, step-potential chronoamperometry, and extinction coefficient of active specie (NiOOH(3+)) for electrocatalysis in (a) 1 M NaOH and (b) 90% CH_3OH on NiOOH electrode.

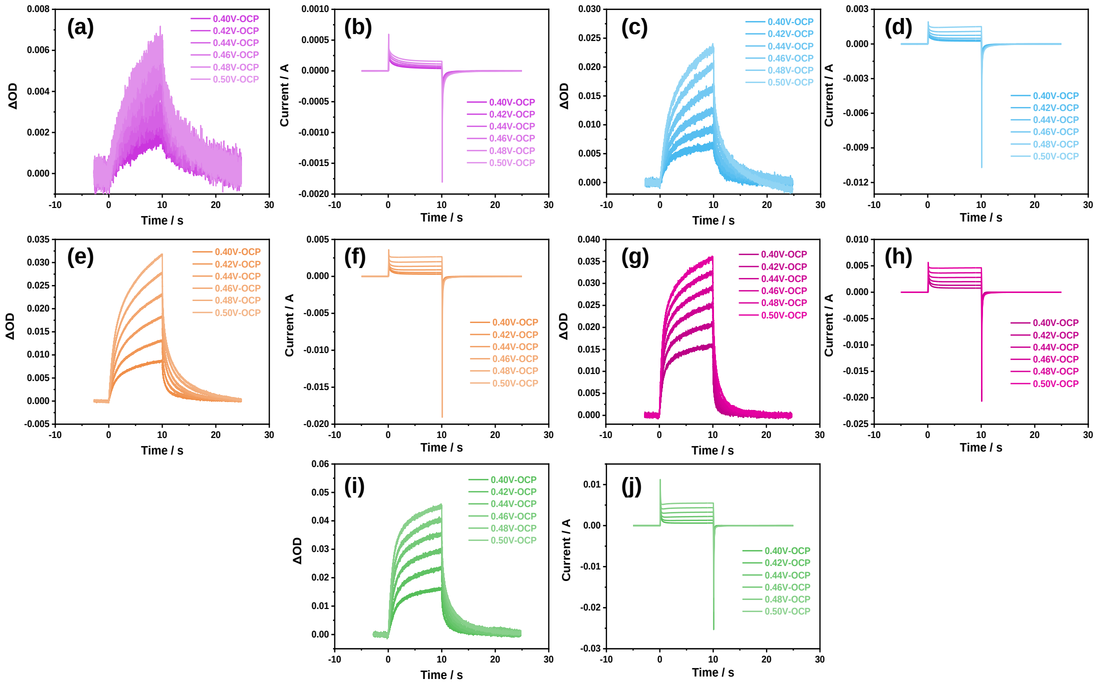

Figure S13. The typical VIA and current data measured during electrocatalytic oxidation of 90% CH_3OH (a, b), 70% CH_3OH (c, d), 50% CH_3OH (e, f), 30% CH_3OH (g, h), and 10% CH_3OH (i, j) by NiOOH(3+) active specie. Probe: 500 nm.

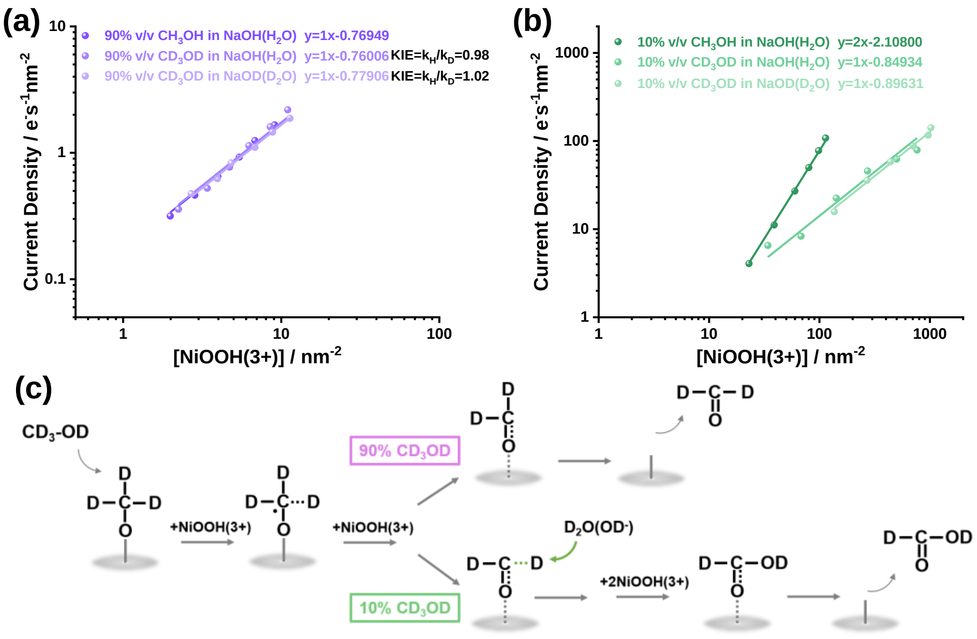

Figure S14. Rate law analysis of (a) 90% CH_3OH oxidation reaction and (b) 10% CH_3OH oxidation reaction by NiOOH(3+) active specie under different deuterated reagents conditions. (c) Schematic representation of perdeuterated methanol oxidation reaction pathway.

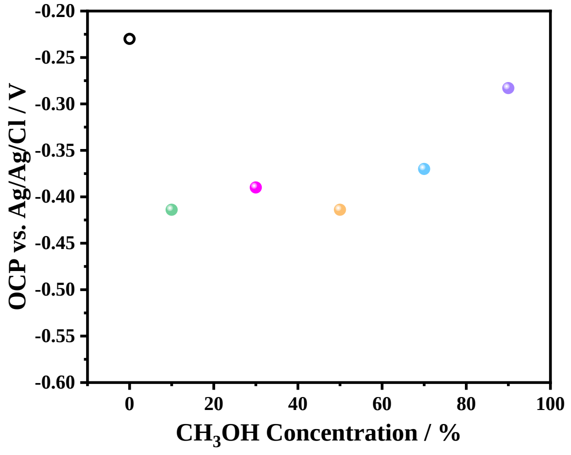

Figure S15. Open circuit potential of the NiOOH electrode as a function methanol concentration. The open circle at 0 % methanol is the OCP measured in 1 M NaOH as the reference.

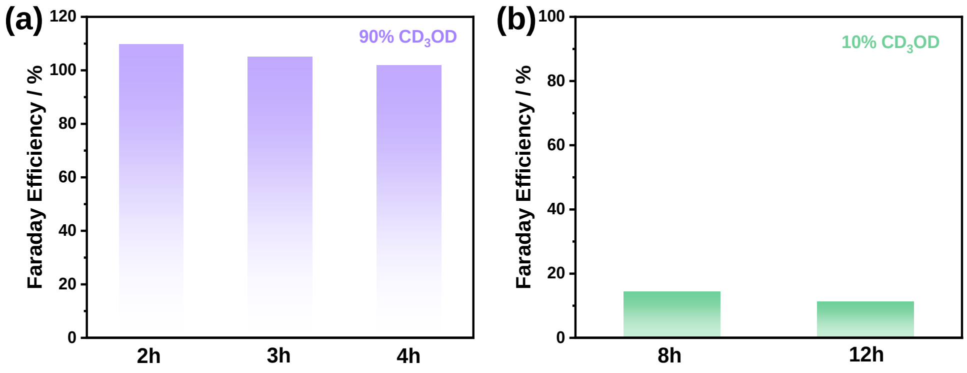

Figure S16. Faraday efficiency of (a) 90% CD_3OD and (b) 10% CD_3OD oxidation to formaldehyde-d_2 at 0.45 $V_{Ag/AgCl}$.

References

1. L. Francas, S. Corby, S. Selim, D. Lee, C. A. Mesa, R. Godin, E. Pastor, I. E. L. Stephens, K. S. Choi and J. R. Durrant, Spectroelectrochemical study of water oxidation on nickel and iron oxyhydroxide electrocatalysts, *Nat. Commun.*, 2019, **10**, 5208.
2. Y. Yuan, Y. Guo, W. Wu, Z. Mao, H. Xu and Y. Ma, Kinetics of active oxide species derived from a metallic nickel surface for efficient electrocatalytic water oxidation, *ACS Energy Lett.*, 2022, **7**, 3276-3285.
3. Z. Ma, X. Guo, Y. Yuan, Y. Wu, Y. Hai, J. Wu, W. Wu and Y. Ma, Operando spectroelectrochemical insights into the selectivity difference of photoelectrocatalytic alcohol oxidation to aldehyde on hematite and titania, *J. Catal.*, 2024, **437**, 115661.
4. J. Yuan, Y. Yuan, J. Zhang, H. Xu, Z. Mao and Y. Ma, Mechanistic insights into selective acetaldehyde formation from ethanol oxidation on hematite photoanodes by operando spectroelectrochemistry, *ChemSusChem*, 2022, **15**, e202102313.
5. C. A. Mesa, A. Kafizas, L. Francas, S. R. Pendlebury, E. Pastor, Y. Ma, F. Le Formal, M. T. Mayer, M. Gratzel and J. R. Durrant, Kinetics of photoelectrochemical oxidation of methanol on hematite photoanodes, *J. Am. Chem. Soc.*, 2017, **139**, 11537-11543.
6. N. W. Jacobsen and R. G. Dickinson, Spectrometric assay of aldehydes as 6-mercapto-3-substituted-s-trizolo(4,3-b)-tetrazines, *Anal. Chem.*, 1974, **46**, 298-299.
7. Y. Yan, J. Zhong, R. Wang, S. Yan and Z. Zou, Trivalent nickel-catalyzing electroconversion of alcohols to carboxylic acids, *J. Am. Chem. Soc.*, 2024, **146**, 4814-4821.
8. Y. Zhang, Z. Lin, S. Duan, Y. Yuan, A. Song and Y. Ma, Spectroelectrochemical identification of CuO impurities catalyzing water reduction on $CuBi_2O_4$ photocathodes, *ACS Energy Lett.*, 2024, **9**, 1761-1770.
